# Pregnancy intention screening tools: a randomized trial to assess perceived helpfulness with communication about reproductive goals

**DOI:** 10.1186/s40834-018-0074-9

**Published:** 2018-12-17

**Authors:** Maureen K. Baldwin, Patricia Overcarsh, Ashlesha Patel, Lindsay Zimmerman, Alison Edelman

**Affiliations:** 1 Department of Obstetrics & Gynecology, Oregon Health & Science University, 3181 SW Sam Jackson Park Road, Portland, OR 97239 USA; 2 Division of Family Planning, Department of Obstetrics and Gynecology, John H Stroger Jr., Hospital of Cook County, 1969 Ogden Ave, Chicago, IL 60612 USA

**Keywords:** Family planning, Reproductive life plan, Counseling tool, One key question®

## Abstract

**Background:**

Federal and clinical guidelines support integration of reproductive life planning in the care of female patients to aid in the reduction of unplanned pregnancies. A multitude of tools have been created to help in the counseling component, but further research is needed regarding how and whether they facilitate patient-provider communication.

**Research:**

We performed a randomized controlled trial to evaluate if patients report whether a detailed or simple pregnancy intention screening tool is helpful for communication of reproductive life plans. We compared a novel reproductive counseling aid, the Family Planning Quotient (FPQ), to a simple tool based on the One Key Question® (OKQ). Providers also evaluated whether they thought the tool used at the visit was helpful. We randomized 93 patients to complete a survey including identical demographic questions and either the FPQ or OKQ reproductive counseling tool. We did not provide further instructions to either the patient or provider. Following the visits, we collected 84 subject evaluations and 79 provider evaluations. A similar proportion of subjects using either reproductive counseling tool found it helpful in communicating their reproductive life plans to their providers (approximately 66%), but there was no difference between the two tools studied. Less than half of providers reported that the FPQ tool was helpful (FPQ: 16/43, 37.2% versus OKQ: 18/36, 50%; *p* = 0.25).

**Conclusion:**

Two-thirds of patients reported either a detailed or simple reproductive plan screening tool was helpful to facilitate communication with their provider, but only half of providers found either tool helpful. Use of reproductive screening tools should be followed by patient-centered counseling to help patients meet their reproductive life goals.

## Background

Unintended pregnancies pose a significant public health burden, so improvements in contraceptive counseling and care aim to improve fertility awareness [1]. Current programs attempt to assess pregnancy intendedness and then either improve contraceptive provision to prevent pregnancy or aid with planning for a healthy pregnancy [2]. Various organizations have developed tools to aid in the counseling component [3–5], but it is not understood if these tools actually work to facilitate efficient patient-provider communication or result in decreased unintended pregnancy rates and healthier pregnancies.

Researchers at John H. Stroger, Jr. Hospital of Cook County developed a unique tool to aid in reproductive-life planning. The ‘Family Planning Quotient’ (FPQ) tool includes a visual and quantitative measurement [4]. The FPQ is defined as a ratio of the number of current children over the number of desired children. The FPQ utilizes a patient worksheet for tracking past pregnancies and future fertility. It also includes an algorithm designed to focus counseling specific to a patient’s goals.

The One Key Question® is a screening question designed to be used in primary care clinic settings. The question is: “*Would you like to become pregnant in the next year?”* [5, 6]. Response options include: Unsure, OK either way, Yes, and No. Depending on the answer, it should be followed by either contraceptive counseling and/or preconception care. We used a simplified screening tool based on One Key Question® (OKQ) with only bivariate responses (Yes, No) to be able to assess a very basic screening tool.

This study aimed to compare patient and provider evaluations of the detailed FPQ versus the basic OKQ tool; specifically to assess patient-provider communication using the tools. We hypothesized that patients would find the FPQ tool helpful to convey their reproductive goals to their providers based on high patient satisfaction in similar studies assessing a communication tool [3]. In contrast, we hypothesized that providers would find the FPQ tool to be unhelpful to their counseling, based on the additional time required.

## A randomized trial to compare the clinical tools

We conducted a randomized controlled trial at Oregon Health & Science University (OHSU) in Portland, Oregon from December 2015 to May 2016. The OHSU Institutional Review Board approved the study protocol and approved this study for a Waiver of Authorization and did not require written consent.

We recruited reproductive-aged (12–45 years old) females presenting to the OHSU Center for Women’s Health for any type of visit but excluded patients who had undergone a prior permanent contraception procedure or had a diagnosis of premature ovarian failure. We did not specify gender identity for inclusion. We included any provider type that might see patients at this outpatient women’s center, including midwives, nurse practitioners, and physicians. These visit types may include routine women’s health maintenance exams by primary care providers (Internal Medicine, Family Medicine, but primarily Ob/Gyn). We performed a simple randomization scheme via a computer generated program with an allocation proportion of 1:1. Surveys were sequentially numbered and placed in opaque sealed envelopes. Verbal assent for enrollment was obtained and patient participants were given the allocated pre-visit survey (FPQ or OKQ) to complete prior to the physician encounter. Patients were instructed to share the survey with their provider. The provider conducted the appointment as usual, using the survey at their discretion. Immediately following the office visit, patients and providers completed a post-visit evaluation. All data was collected without identifiers. Participants could withdraw from the study at any point.

Both surveys included ten identical demographic questions. The FPQ survey included the unique question, “*How many children would you have liked or would you like to have in your family*?” and asked for more details about each past pregnancy such as intentions and outcomes. It also elicited a more detailed response to plans for future pregnancies by having participants choose from the following: I am trying now; I am trying in 1–2, 3–5, or 5 or more years to have a baby; I do not want more children; or I am not sure. The OKQ survey included only one unique question, “*Would you like to become pregnant in the next year?”* [5]. We only allowed a bivariate Yes/No answer to assess the administration of a very basic screening tool compared to the more detailed one. Neither survey asked about contraceptive practices, infertility, or fertility preferences of a partner.

The primary outcome was the patient’s response to the question, “*This tool helped me to communicate my own personal goals to my provider.*” Additionally, we compared the response by providers to the question, “*This tool helped me to focus the counseling I provided to my patient.*” Responses were reported on a 5-point Likert scale (1 = strongly agree, 5 = strongly disagree) and then dichotomized to agree versus neutral or disagree.

We estimated that 80% of patient participants and 30% of their providers would find the FPQ tool helpful in communicating or reviewing reproductive goals. Therefore, we planned to enroll at least 16 subjects in each group to demonstrate a clinically important difference between the tools of at least 50% with power of 90% and an alpha of 0.05 using a two-sided chi-square test of proportions. To be able to analyze a comparison of provider rating, we planned to have a minimum of 36 completed provider evaluations in each group. We performed descriptive statistics for baseline characteristics of the two groups.

## Results of the randomized trial

**Fig. 1:**
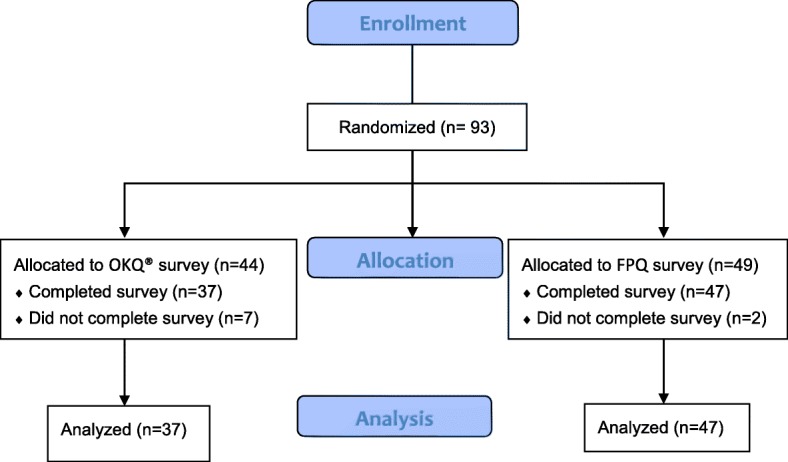
Consort diagram

A total of 93 survey packets were distributed and 89 patient participants completed the pre-visit survey. Seventy-six patients (FPQ *n* = 39, OKQ *n* = 37) and 79 providers (FPQ *n* = 43, OKQ *n* = 36) completed post-visit evaluations (Fig. 1). The average subject was a 28-year-old white female (age range 17–40) with some college education or higher with public insurance (Table 1). Only three of 39 subjects (7.7%) had a calculated FPQ greater than 1 indicating that they had more children than they desired. The majority of subjects in the FPQ group wanted more children than they currently had (31/39, 79.5%). In contrast, 34/39 (87.2%) participants in the OKQ group reported they did not want to become pregnant in the next year (17/20, 85% of currently non-pregnant).

**Table 1:** Baseline demographics compared between women’s health clinic patients randomized to complete a novel Family Planning Quotient tool versus the modified One Key Question®

	FPQ (*n* = 48) *n* (%)	OKQ (*n* = 43) *n* (%)
Age (mean ± SD)	28 ± 6.5	29 ± 5.4
Race		
White	33 (68.8)	30 (69.8)
Black	1 (2.1)	5 (11.6)
Other	14 (29.1)	8 (18.6)
Education		
High school or less	12 (25.0)	6 (14.0)
Some college	17 (35.4)	19 (44.2)
College or more	19 (39.6)	18 (41.9)
Income		
< $30,000	23 (48.9)	21 (51.2)
$30–59,999	13 (27.7)	8 (19.5)
$60,000+	11 (23.4)	12 (29.3)
Insurance status		
Private	17 (35.4)	8 (20.0)
Public	31 (64.6)	31 (77.5)
Uninsured	0	1 (2.5)
Relationship status		
Single	8 (16.7)	8 (18.6)
Dating/In a relationship	18 (37.5)	15 (34.9)
Living together	8 (16.7)	6 (14.0)
Married	12 (25.0)	13 (30.2)
Divorced/Separated	2 (4.1)	1 (2.3)
Sexual partners		
Male	40 (83.3)	35 (81.4)
Female	0	0
Both	0	1 (2.3)
Not currently sexually active	8 (16.7)	7 (16.3)
Total number of children (biologic and non-biologic)		
None	20 (41.7)	22 (52.3)
1–2	20 (41.7)	11 (26.2)
3 or more	8 (16.7)	9 (21.4)
Currently pregnant	19 (39.6)	22 (51.2)

After the visit, about two-thirds of patients in both groups reported that the survey tools were helpful in communicating their reproductive goals to providers (FPQ 31/47, 66%; OKQ 25/37, 67.6%; *p* = 0.88). Fewer providers agreed the FPQ tool helped to focus their counseling (FPQ 16/43, 37.2% versus OKQ 18/36, 50%; *p* = 0.25). Additional patient responses to other survey questions about communicating reproductive goals with a provider demonstrated that more patient participants in the FPQ group (35/46, 76.1%) compared to the control group (19/37, 51.4%) agreed with the statement: “*Overall, this tool is helpful and I would use it to track my reproductive health goals*” (*p* = 0.02).

## Commentary

Reproductive counseling aids, such as the two studied here, may provide valuable screening or tracking as reproductive goals evolve over a woman’s lifetime. However, even if they are perceived as helpful communication tools by the patient and/or their provider, their utility depends on the larger picture of whether the patient was able to develop and achieve her goals. However, a patient’s reproductive goals are likely much more complicated than the binary information or a ratio derived from these tools. For example, a plan to have three children might be adjusted after the first two. A desire for another baby might be in conflict with a partner’s plans or might be impacted by infertility or lack of a partner. It is unknown if either of these tools has an impact on overall community health outcomes like decreasing unplanned pregnancy rates or rapid repeat pregnancies.

As other researchers have discussed, use of a value-based measure like “planned pregnancy” also assumes that women will respond to a binary description of pregnancy intention and will be receptive to preconception counseling [7, 8]. In fact, the current version of the One Key Question® is designed to have four possible outcomes (Unsure, OK either way, Yes, and No) to the question, “*Would you like to be pregnant in the next year?*” We only allowed a binary Yes/No response for this study in the interest of simplicity. It is possible that with the expanded multiple options, either patients or providers might have rated the tool as more helpful. The main benefit of any reproductive screening tool is to raise the topic of fertility awareness for discussion in environments where it has been an afterthought. These study findings are limited by a clinical environment amenable to a discussion about fertility goals. Another limitation is that we did not record the extent to which the clinician reviewed or used the tools. Additionally, we have notstudied the impact or quality of the counseling that might have occurred after use of the screening tool. While this patient population was representative of our clinic demographic, the population was primarily made up of white educated women, which limits generalizability. While subjects in the FPQ group showed a preference for tracking their goals with the FPQ, they were more likely to have plans to pursue pregnancy in the future than those in the OKQ group. We do not think this significantly affected the study outcome, since they responded similarly to other questions about whether the tool helped to communicate or focus the conversation.

Patient-centered counseling including open-ended questions, collaboration to identify strategies, acceptance of ambivalence and unintended pregnancy, and tailored nonjudgmental counseling is proposed as a way to help patients identify and meet reproductive goals [8]. However, patient-centered counseling takes time and thus, can be difficult to integrate into a clinical encounter. In choosing a counseling tool, we think that patients like a complex and structured format, perhaps one that allows for ambiguity: whereas providers might appreciate brevity and information that requires immediate action. To fully assess the utility of such a tool for a provider, it should be tested in an environment where fertility goals may be a secondary concern.

Future research and quality performance measures should focus on the proportion of health screenings that address and document patient-centered counseling about reproductive goals, and should attempt to assess whether counseling and documentation takes excess time, and whether discussion of goals results in increased access to services and improved health outcomes in the population.

## Conclusions

Participants exposed to either a simple or complex reproductive screening tool equally reported that the tool helped them to communicate their reproductive goal to their providers, but providers reported that the tools were not particularly helpful. Patients may appreciate a way to broach this topic with their care providers and it may not matter which tool is used. Future research should focus on whether screening tools correlate with improved health outcomes. Given time constraints in clinic, any job aid needs to be easy to integrate and efficient, and should provide enough information to facilitate individualized counseling.

## Abbreviations

FPQ: family planning quotient
OKQ: simplified screening tool based on One Key Question®
